# Supplementary material for: Exploring the use of self-management strategies in survivors of critical illness: A scoping review protocol
Source: PLoS One. 2026 Apr 30;21(4):e0347646. doi: 10.1371/journal.pone.0347646 (PMC13132182; doi:10.1371/journal.pone.0347646)
Supplement: S1 Appendix — (DOCX) [file pone.0347646.s001.docx]

**S1 Appendix: Preliminary Search Strategy**

**Medline Search Strategy**

| **Concept** | **Search Terms** |
| --- | --- |
| Intensive Care | 1. intensive care units/ or burn units/ or coronary care units/ or respiratory care units/  2. (intensive care or burn unit* or coronary care unit* or respiratory care unit* or ICU or ICUs).mp.  3. Critical Illness/  4. critical* ill*.mp.  5. Critical Care/  6. critical care.mp.  7. airway management/ or airway extubation/ or intubation, intratracheal/ or respiration, artificial/ or ventilator weaning/ or tracheostomy/  8. (airway management or extubat* or intubat* or ventilator* or mechanical* ventilat* or tracheostomy or artificial respiration).mp.  9. Respiratory Insufficiency/  10. (respirat* insufficiency or respirat* failure).mp.  11. post-intensive care syndrome.mp.  12. PICS.mp.  13. exp animals/ not humans.sh.  14. 1 or 2 or 3 or 4 or 5 or 6 or 7 or 8 or 9 or 10 or 11 or 12 or 13 |
| Self-Management | 15. exp Self Care/  16. exp Self-Management/  17. exp Patient Education as Topic/  18. exp Social Support/  19. exp Behavior Therapy/  20. exp Goal/  21. exp Problem Solving/  22. exp Medication Adherence/  23. self?management.mp.  24. self?care.mp.  25. self?help.mp.  26. coping strateg*.mp.  27. patient education.mp.  28. empower*.mp.  29. self?efficacy.mp.  30. self?guided.mp.  31. goal setting.mp.  32. medication management.mp.  33. symptom self-management.mp.  34. social support.mp.  35. behavio?r change.mp.  36. problem?solv*.mp.  37. medication adherence.mp.  38. 15 or 16 or 17 or 18 or 19 or 20 or 21 or 22 or 23 or 24 or 25 or 26 or 27 or 28 or 29 or 30 or 31 or 32 or 33 or 34 or 35 or 36 or 37 |
| Post-Hospital | 39. post-hospitalization.mp.  40. post-hospitalisation.mp.  41. post recovery.mp.  42. post critical care.mp.  43. post ICU.mp.  44. post hospital.mp.  45. patient discharge.mp.  46. after illness.mp.  47. exp Treatment Outcome/  48. community.mp.  49. 39 or 40 or 41 or 42 or 43 or 44 or 45 or 46 or 47 or 48 |
|  | 14 AND 38 AND 49 |
